# “And now I just have to disappear”: A Gerontological Reading of *The Last Showgirl*

**DOI:** 10.1093/geront/gnaf146

**Published:** 2025-05-30

**Authors:** Ronald W Berkowsky

**Affiliations:** Department of Health Science, California State University Channel Islands University Dr., Camarillo, CA 93012, USA

**Film**: *The Last Showgirl* (88 min)

**Director**: Gia Coppola

**Released**: January 10, 2025 (USA—wide)

**Distributor**: Roadside Attractions (USA)

**Figure 1.**
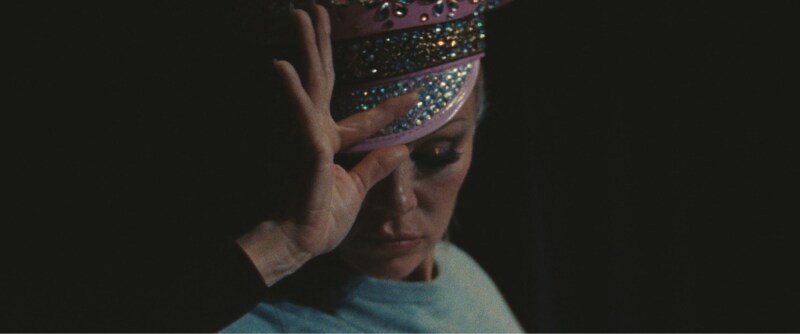
Pamela Anderson as “Shelly” in *The Last Showgirl* (Coppola, 2024). Image reproduced with permission of Mongrel Media.

In the opening scene of Gia Coppola’s *The Last Showgirl* (2024), Shelly (Pamela Anderson) takes to an audition stage (see Figure 1). The Las Vegas revue to which Shelly has dedicated her career, *Le Razzle Dazzle*, is ending—and she is begrudgingly in search for a new show to express her creativity while remaining gainfully employed. While on stage, an unseen casting director (Jason Schwartzman) probes Shelly for background—name, height, and age. At this last inquiry, Shelly responds with an awkward playfulness (“Oh, a gentleman never asks a lady her age!”) before answering that she is 36—only to promptly apologize for lying and correcting her response to 42 (she is, in fact, 57). She proceeds to qualify this response by joking, “This house is *huge*, distance helps,” signaling to the casting director that her age will *not* be a problem—the performance venue is large enough that, of course, the audience will be far enough away that nobody will be able to *tell* she is 42 (or 57). This brief exchange and the preceding moments underscore the intersection of themes at the heart of Coppola’s film: the search for purpose and meaning during major life transitions, the struggle for financial security among aging adults, and the compounded impacts of ageism and sexism (i.e., women in Shelly’s line of work *must* be young and they *must* adhere to a particular beauty standard, all others need not apply). At a crisp 88 min, Coppola’s film, anchored by an impeccably heartbreaking lead turn by Anderson, spares no time in its exploration and interrogation of aging, resilience, and societal attitudes toward aging women.

A central theme of *The Last Showgirl* is its exploration of work and financial security (or insecurity) among older women. Las Vegas is portrayed as a city whose industries thrive on ageist standards of beauty—not just in entertainment, but in service as well. This is apparent in scenes featuring Shelly’s friend Annette (Jamie Lee Curtis), a casino cocktail waitress who, when her manager informs her that they are “cutting the floor” (e.g., reducing service staff during nonpeak hours), vents frustration at being asked to leave when her younger coworkers remain. While Annette’s age is never revealed, we can presume she is in her late 60s (Curtis would have been 65 around the time of filming). She is repeatedly exasperated at the way in which she is treated because of her age but continues working in an industry that does not value her as it does others. This is because employment for Annette is not an avenue for personal growth or meaning per se—for her, employment is *essential* and a means for survival. This is highlighted in one of the most cutting lines delivered by Curtis early in the film, in response to a question on when she will retire:

*“Retire? Are you crazy? Like, bankers retire. I – what do you think, I have a 401(k)? No, I’m not gonna retire. I’m not gonna retire. I’m gonna work, and then I’m gonna work some more, and then I’m gonna die. I’ll probably die in my uniform. That’s my plan…You know, it’s not an option…”*

This highlights a reality for many older adults: that the pursuit of financial security in old age often precludes one from being able to safely navigate from work life. Recent analyses by the National Council on Aging, in partnership with the LeadingAge LTSS Center at UMass Boston, provide real-world context to Annette’s predicament: it is estimated that 80% of US adults aged 60+ currently experience financial insecurity, and nearly half of all older adults have incomes below what they need to cover basic necessities (Tavares and Glova, 2024). Annette is not alone in her struggles, as Shelly also faces economic precarity in an industry where wages fluctuate and pensions are rare. In a scene where Shelly is docked pay due to a rip in one of her costumes (i.e., a “wing” accidentally torn on a much-maligned *new* door handle) that needs mending, she expresses disappointment to *Le Razzle Dazzle*’s stage manager Eddie (Dave Bautista) as she admits that she did not budget for a paycheck so small.

This ripped wing also serves as a metaphor for Shelly herself: elegant, flashy, glitzy, but broken and at the brink of being completely discarded. Shelly is aware that her time at *Le Razzle Dazzle* is limited, and she is unsure if she will be able to find comparable, meaningful work in an industry which privileges youth. This highlights another major theme of the film: the search for purpose and meaning in later life. Throughout the film, Shelly repeatedly shares stories and perspectives which underscore how attached she is to her work and to *Le Razzle Dazzle*—the show defined her career, and her career defined her persona. She is now in dire fear of losing her identity and of being discarded or forgotten, as evidenced by an exchange with Eddie: “I love the show. I love it. I feel so good about myself in the show…and now I just have to disappear.” Regardless of the many financial considerations, retirement never appears to be an option for Shelly. Retirement is often framed in public discourse as a period of leisure and reflection, but scholars have long highlighted the process of retirement as a significant and potentially stressful life event (Holcomb, 2010). While retirement can be a positive experience and an opportunity for growth for those leaving dissatisfying jobs, the transition away from work life for those whose identities are deeply tied to their employment can contribute to declines in purpose (e.g., Yemiscigil et al., 2021). It is clear that Shelly is not ready to abandon the work which brings her meaning—but as she struggles to secure another show, her behavior and outbursts illustrate a crisis of identity.

Beyond a simple exploration of ageism, Coppola’s film also addresses the unequal pressures that shape women’s choices through the life course. This is most evident in examining the relationship between Shelly and her daughter Hannah (Billie Lourd). While Shelly is proud of her work with *Le Razzle Dazzle* and argues that it has brought her life meaning and purpose, Hannah argues that this devotion was at the expense of her childhood, noting that she was often left to her own devices (in the car in a hotel parking lot, no less) while her mother was on stage. When Shelly broaches the topic of her strained mother-daughter relationship to Eddie late in the film, he suggests to her that she could have considered a different career to allow her more time with Hannah. Shelly is quick to berate Eddie for this suggestion as it is emblematic of the still-prevailing view of women as caregivers and not breadwinners. While scholars have noted gradual attitudinal changes toward the roles of women in the workforce (Scott and Braun, 2009), there is still evidence that women are judged and penalized for violating the gendered norms surrounding work and home life (e.g., Gaunt, 2012). Shelly does not completely absolve herself of the consequences of her choices—she remains steadfast in her commitment to *Le Razzle Dazzle* but is frequently distraught over the state of her relationship with Hannah—but the film carefully underscores the unique societal judgments and pressures working mothers endure, particularly those pursuing more artistic careers.

It is key that Coppola explores the complexities of ageism, sexism, financial security, and resilience against the backdrop of Las Vegas. Despite the sparkling exterior, the city has long served as a metaphor for the impermanence of success and youth. The film is littered with scenes of Shelly walking or dancing the streets adjacent to recognizable landmarks, hotels, and casinos, but these strolls are notable in that the streets appear mostly *empty*. A similar scene occurs mid-film in a sequence focused on Hannah, also seen walking the streets of Las Vegas. In her stroll, she passes by the ruins of the newly demolished Tropicana, a subtle nod to the city’s pursuit of newer and more modern attractions (at the expense of the classic, or the aged). The struggle of the city parallels Shelly’s own.

Whereas recent films such as the Academy Award-nominated *The Substance* (Fargeat, 2024) also interrogate themes of aging in youth-centered culture, *The Last Showgirl* is far more measured, straightforward, and classical than Fargeat’s satisfyingly gonzo approach. This is not a criticism of Coppola’s work—in fact, it is quite refreshing to see a more subdued (yet still, in its own way, glamorous) portrayal of Las Vegas in comparison to the shadier versions we see in films such as *Leaving Las Vegas* (Figgis, 1995) and *Showgirls* (Verhoeven, 1995). Flush with superb turns by a uniformly strong cast, *The Last Showgirl* succinctly and beautifully illustrates how aging adults (and, in particular, aging women) navigate the complexities of work and retirement, self-worth, and maintaining stability and relevance in a city and industry that can be wholly unforgiving.
